# Is the Responsiveness to Light Related to the Differences in Stem Straightness among Populations of *Pinus pinaster*? †

**DOI:** 10.3390/plants8100383

**Published:** 2019-09-28

**Authors:** Rosario Sierra-de-Grado, Valentín Pando, Pablo Martínez-Zurimendi, Bruno Moulia

**Affiliations:** 1 Sustainable Forest Management Research Institute University of Valladolid, Avda de Madrid 44, 3004 Palencia, Spain; rsierra@pvs.uva.es (R.S.-d.-G.); vpando@eio.uva.es (V.P.); 2 Departamento de Agricultura, Sociedad y Ambiente, El Colegio de la Frontera Sur, Unidad Villahermosa 86280, Mexico; 3 UCA, INRA, UMR PIAF, 63000 Clermont-Ferrand, France; bruno.moulia@clermont.inra.fr

**Keywords:** compression wood, gravitropism, photomorphogenesis, phototropism, stem straightness

## Abstract

Stem straightness is related to wood quality and yield. Although important genetic differences in stem straightness among the natural populations of *Pinus pinaster* are well established, the main drivers of these differences are not well known. Since the responses of trees to light are key ecological features that induce stem curvature, we hypothesized that populations with better straightness should exhibit lower photomorphogenetic and phototropic sensitivity. We compared three populations to identify the main processes driven by primary and secondary growth that explain their differences in response to light. One-year-old seedlings were grown under two treatments—direct sunlight and lateral light plus shade—for a period of 5 months. The length and the leaning of the stems were measured weekly. The asymmetry of radial growth and compression wood (CW) formation were analyzed in cross-sections. We found differences among the populations in photomorphogenetic and phototropic reactions. However, the population with straighter stems was not characterized by reduced sensitivity to light. Photo(gravi)tropic responses driven by primary growth and gravitropic responses driven by secondary growth explained the kinetics of the stem leaning and CW pattern. Asymmetric radial growth and CW formation did not contribute to the phototropic reactions.

## 1. Introduction

The shape of tree stems can be affected by many environmental factors during the life of the tree (snow, wind, landslides, lateral incidence of light, damage to the apex, etc.), which can induce curvatures in the stem. Indeed, any increase in tree mass can induce bending movements in the slender structure of tree stems, resulting in notable curvatures [1]. However, conifers also exhibit curvatures driven by compression wood (CW) [1]. This type of curvature allows the tree to achieve a stable conformation but negatively affects the cost of transport, industrial processing, yield in raw material and quality of the final product due to reaction wood formation [2,3]. In conifers, CW appears on the lower side of leaning and/or in the concave side of curved stems regardless of the cause of leaning or curvature. CW formation is frequently associated with eccentric radial growth, longitudinal shrinkage during the drying process and growth stresses due to maturation strains, all of which are detrimental to the industrial use of wood [4]. In species such as *Pinus pinaster* Ait., a major timber species in many countries, the remarkably frequent flexuosity of trunks causes severe economic losses.

In *P. pinaster*, phenotypic and genetic differences in stem straightness among natural populations from different geographic provenances are well known [5,6,7,8]. These differences are pronounced, as the trees range from straight to very twisted. In addition, in provenance trials in different sites—in which plants from different provenances are grown under common environmental conditions—the stem straightness performance of the plants from each population is quite stable and consistent with the straightness in the natural forests of origin [6].

However, the drivers of such differences, whether adaptive or not, remain unclear. In boreal conifers, ecotypic differences in trunk shapes have been associated with adaptations to snow or wind [4], but this type of adaptation cannot explain the great variability of shapes in species such as *P. pinaster* or *Picea abies* [9,10]. Due to the great natural geographical variability in *P. pinaster*, the comparison of trees from different provenances in a common environment may be useful to identify the underlying drivers that explain these differences.

One external factor that affects stem straightness is the lateral incidence of light. Differences between species in their responses to lateral light and in their reorientation strategies have been reported both in conifers [4,11,12] and in broadleaved species [1,13,14]. Early German works suggested a correlation between the tendency to develop curved stems and the phototropic sensitivity of seedlings in different conifer species (Achterberg 1953, Lyr et al. 1967 and Mayer 1964 cited in Timell [4]). Different sensitivities to light could also explain genetic differences in straightness among populations.

In the forest, different directions of light and variations in its quality can affect the growth and form of young trees, e.g., when gaps are opened in a dense canopy or when the density of neighboring plants varies [13,15]. Plants grow toward the lateral light by curving the growing stem. This reaction is considered a phototropic response that improves the plant’s ability to collect light and hence plays a major ecological role [16,17,18]. If the plant is under canopy shade or surrounded by other vegetation, variations in the light quality induce photomorphogenetic responses, which are typically characterized by an increase in the height and slenderness of the stems and a decrease in branch and root biomass, known as shade avoidance syndrome (SAS) [16].

Phototropic responses in plants are linked to complex molecular, biochemical, and cellular processes associated with the detection of the direction and quality of light and with signaling pathways. Comprehensive reviews has been published by Liscum et al. [19], Fankhauser and Christie [20] and Fiorucci et al. [15]. The detection of directional blue light occurs both in the plasma membrane and in the cytoplasm and nucleus, involving at least six photoreceptors. Light cues induce a lateral gradient of auxin, increasing the auxin concentration in the shaded side compared to the lit side, which leads to the bending of stems toward the light in the phototropic response. Multiple proteins (e.g., phot1/phot2, NPH3, RPT2), nine auxin transporters, seven auxin receptors, multiple transcriptional regulator target genes and four phytohormone signaling pathways (auxin, ethylene, BR, and gibberellin) are involved in the responses of the plant to directional light [19].

Under a canopy, the light is filtered by foliar layers, which preferentially absorb the red and blue light wavebands, decreasing the red/far-red ratio (R:FR) and the radiance of blue light. Phytochrome photoreceptors (phy), cryptochrome blue light photoreceptors (cry) and PIF transcription factors play a major role in detecting changing light intensities and spectral variations, thereby leading to SAS responses [21,22]. Plant species are classically classified as shade tolerant or shade avoiding according to their response to shade. Inter- and intraspecific variations in shade responses have been demonstrated in several groups of plants, and particularly variation associated with the environmental conditions of the geographical origin of the populations [23,24,25,26].

When the vertical growth direction of a stem deviates toward lateral light, a change in its orientation relative to the gravitational field is produced. Then, in addition to the phototropic response, a gravitropic reaction is elicited [14,27], unless a gravitropic correction occurs, such a plant become increasingly mechanically compromised as it grows due to the increased risk of bending and buckling under its own weight [1,14,28].

All these reactions cause curvatures in the stem that, in turn, are sensed through proprioception [27,29] and elicit a contra-curvature response (autotropic or autostraightening reaction) [27,29,30]. Finally, the balance between the sensing of these three drivers (photosensing of lateral light, gravisensing of the inclination and proprioceptive sensing of the stem curvature) controls the final straightness of the stem [31].

The efficiency of achieving shape control also depends on the motors involved in the curvature of the stem [30,32], which may differ depending on the type of stimulus. When the stimulus is gravitropic (stem tilting), two different motors act on different parts of the stem. Firstly, a rapid reaction driven by primary growth (onward primary reaction) causes the apex to curve upward in the vertical direction due to the differential elongation of the cells on the upper and lower sides of the inclined stem. This primary gravitropic reaction is considered widespread in plants, although it does not appear to be present in *Pinus taeda* [33]. Secondly, a slower reaction driven by secondary growth (onward secondary reaction) involves asymmetric radial growth, the formation of CW and a resulting asymmetry of maturation strains and growth stresses [34]. This asymmetry of stresses between opposite sides of a section of the stem produces an active change in curvature [35]. All these curves, in turn, provoke a countercurvature response [36], which in the case of *P. pinaster* involves the development of CW [32].

When the stimulus is phototropic, differential cell elongation on the illuminated and shaded sides of a stem also causes a primary phototropic curvature [19], but little is known about the secondary reactions in response to light. Matsuzaki et al. [37] suggested that asymmetrical xylem formation (both normal and tension wood) may be the mechanism responsible for phototropic bending in nonelongating but radially growing stems of *Quercus crispula* seedlings, but to our knowledge, this explanation has not been demonstrated. In conifers, inconclusive reports had suggested that phototropic stimulus was responsible for an increase in the % CW area in unthinned stands [38,39], but the role of CW within the phototropic reactions remained unaddressed, as the directionality of the stimulus respect to the CW formation had not been considered. Since phototropism and gravitropism are not easy to disentangle [14], both effects can be mixed.

In this work, we induced photomorphogenetic and photogravitropic responses in seedlings from three different geographic provenances of *P. pinaster* that differ in their stem straightness and growth: The Sierra de Gredos population, with rapid growth and very straight stems; Bajo Tiétar, with tall trees but sinuous stems; and Sierra de Oña, with slow growth and crooked stems (Gredos, Tiétar and Oña onward). Two contrasting treatments were established: Direct sunlight (DLT) and lateral light plus deep shade (LLT). In the LLT treatment, each plant was covered with a tunnel with a double 80% black shade cloth, leaving open only the south side to induce a phototropic curvature and achieve a drastic reduction in the light intensity.

Our aims were as follows: 1) to test whether differences in responsiveness to light conditions (shade and lateral incidence) are associated with differences in stem straightness among populations; if so, then populations with better straightness should exhibit a lower photomorphogenetic response (less elongation) or lower phototropic sensitivity (less deviation in response to the same lateral light stimulus) and 2) to clarify the role of secondary reactions (asymmetric radial growth and CW formation) in phototropic responses. We hypothesized that the populations with straighter genotypes would be less sensitive to lateral light and produce less CW.

Our results revealed important intraspecific variability in photomorphogenetic and phototropic responsiveness, although the straight-stemmed population was not characterized by low responsiveness. We did not find evidence of secondary phototropic reactions. Asymmetric radial growth and CW formation have been linked to gravitropic reactions but not to photoresponses, at least in relatively young plants.

## 2. Results

**Figure 1:**
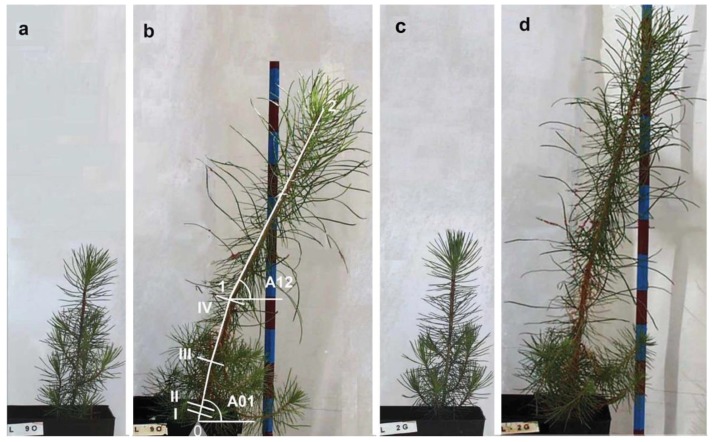
Development of two representative plants under lateral light plus deep shade (LLT): A plant from Oña provenance at the beginning (**a**), and at the end of the experiment (**b**) and a plant from Gredos provenance at the beginning (**c**) and at the end (**d**) of the experiment. The pictures were taken in the north-south plane, with lateral light coming from the south (right side of the pictures). In (**b**), the division of the stem in segment 01 developed during the first growing season of the plant, and segment 12 developed during the experiment; the angles measured in the plants (A01 and A12) and the locations of the analyzed cross-sections (I to IV) are shown.

The stems were considered in two parts (Figure 1): The basal part, which developed during the first year of plant growth (segment 01), and the upper part, which developed during the experiment (second growing year, segment 12). The angles of segments 01 and 12 of the stems with respect to the horizontal direction (A01 and A12, respectively) were used to follow the changes in the leaning of the stems. Those angles and the stem length (Li) were measured weekly. Length growth was computed as L_i_−L_0_, with L_i_ being the length i days after the beginning of the experiment and L_0_ the initial length.

### 2.1. Photomorphogenetic Effects

#### 2.1.1. Length Growth

**Figure 2:**
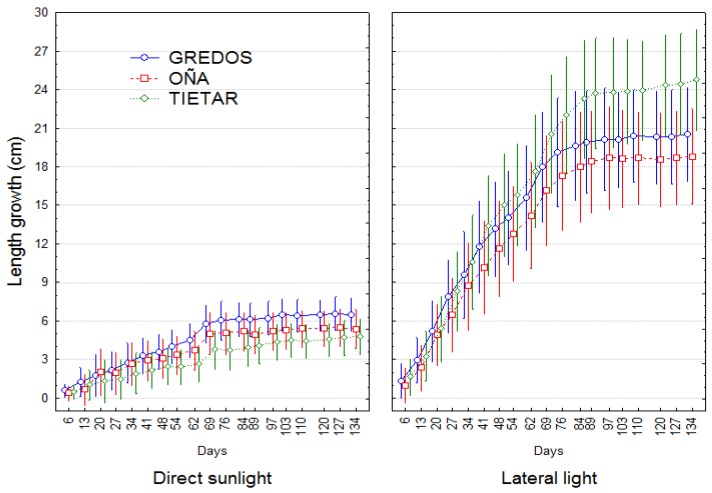
Length growth curves for three populations (Gredos, Oña and Tiétar) undergoing direct sunlight (DLT) and LLT over time. Length growth was computed as L_i_−L_0_, with L_i_ representing the length i days after the beginning of the experiment and L_0_ the initial length. Dots represent least-squares means, and bars represent 95% confidence intervals. Total number of observations n = 48.

The plants stopped elongating earlier under DLT than under LLT (Figure 2). The individual contrasts in length growth between consecutive dates of measurement were significant from the beginning of the experiment to 69 days (July 14th) in DLT and from the beginning to 89 days (August 3rd) in LLT. Subsequently, the plants no longer showed noticeable primary growth until the end of the experiment.

Oña plants were the shortest plants at the beginning of the experiment, exhibiting a significant difference from the Gredos and Tiétar plants (Table 1). Under DLT, there were no significant differences in length growth (Gd) among provenances.

As expected, plants under LLT elongated more than plants in direct sunlight, with a mean of approximately 393% more (Table 1, Figure 2). Under LLT, Tiétar plants grew more than Oña plants. At the end of the experiment, the length growth under LLT compared with the length growth under DLT (Gl−Gd), determined as an estimation of the photomorphogenetic effect, was 49.1% greater in Tiétar than in Oña plants (*p* = 0.0039) and 42.7% greater in Tiétar than in Gredos plants, which was also significant (*p* = 0.024) (Table 1).

#### 2.1.2. Asymmetry of Radial Growth

**Figure 3:**
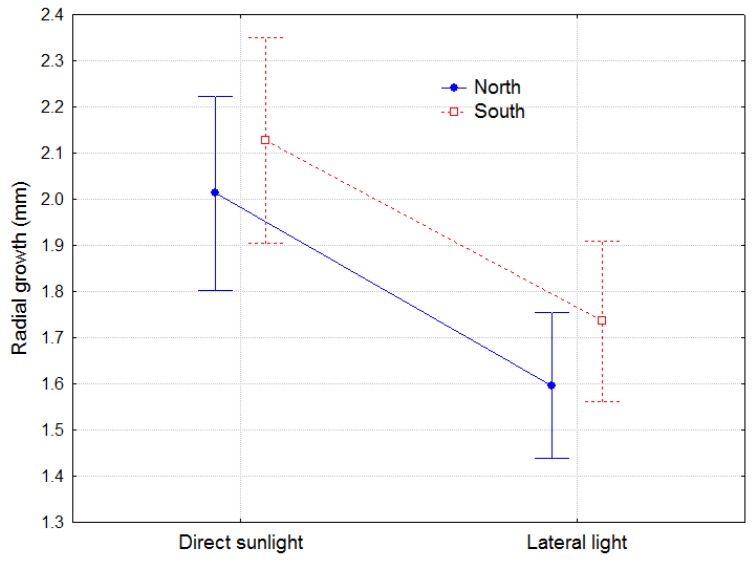
Radial growth on the north and south sides of the stem in cross-section II for DLT and LLT during the experiment. Bars represent 95% confidence intervals. Total number of observations n = 36.

The LLT led to a decrease in radial growth at the base of the epicotyl stem (section II) compared with DLT (Figure 3). The effect of the provenance was not significant. The radial growth was slightly greater on the south side than the north side (mean difference of 0.13 mm, representing 7% more on the south, *p* = 0.0052). The difference between radial growth on both sides was not significant between treatments (*p* = 0.7691).

The asymmetry of the cross-section (ratio between the north-south and east-west diameters of cross-section II) did not show differences between treatments or among provenances.

#### 2.1.3. Slenderness

**Figure 4:**
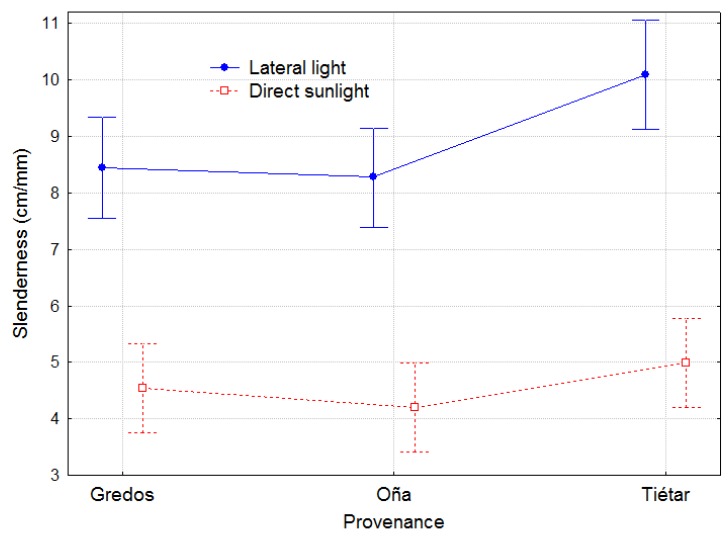
Slenderness (ratio between the final length of the plant and the mean diameter of section II at the end of the experiment) per treatment and provenance. Bars represent 95% confidence intervals. Total number of observations n = 48.

The slenderness was computed as the final length of the plant (cm) divided by the mean total diameter in section II (mm). The slenderness under DLT did not differ among provenances. However, under LLT, the slenderness was almost twice that in DLT (*p* < 0.0100), and the responses among the provenances were diverse (*p* = 0.0005), with Tiétar plants exhibiting the greatest slenderness and significantly distinct behavior from that of the other provenances (Figure 4).

### 2.2. Phototropic and Gravitropic Effects

#### Kinetics: Changes in Angles in the Vertical North-South Plane

**Figure 5:**
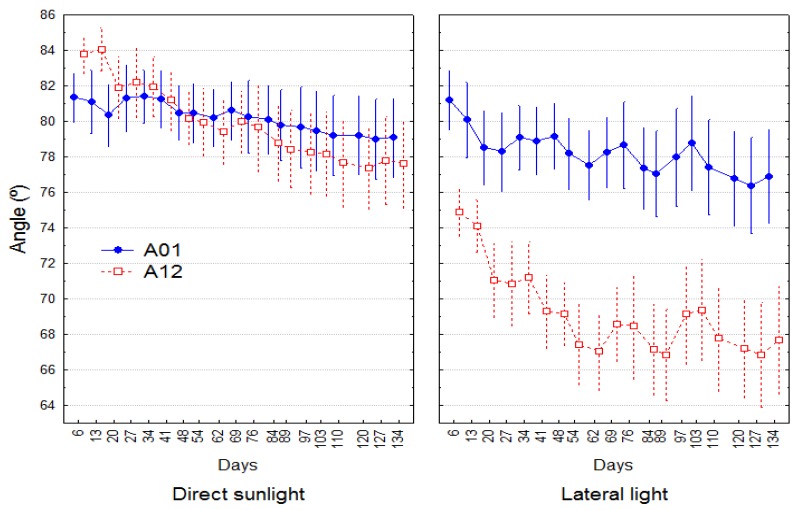
Changes over time of angles A01 and A12 (averaged provenances) under DLT and LLT. Means and their 95% confidence intervals are plotted. Total number of observations n = 48.

The plants did not start from a vertical position due to the initial conditions (see Materials and Methods), but there were no significant differences in the starting angle among provenances. Under DLT, the plants exhibited a stable leaning in the basal part of the stem (A01) and a slightly increased leaning in the upper part (A12) with no significant difference from A01. The LLT significantly affected only the leaning of the elongating part of the stem (A12) (Figure 5). There were no differences between treatments in angle A01.

There were no differences among provenances in the angles under DLT either in the whole experimental period or when splitting it into the elongation and not elongation periods (Table 2). 

**Figure 6:**
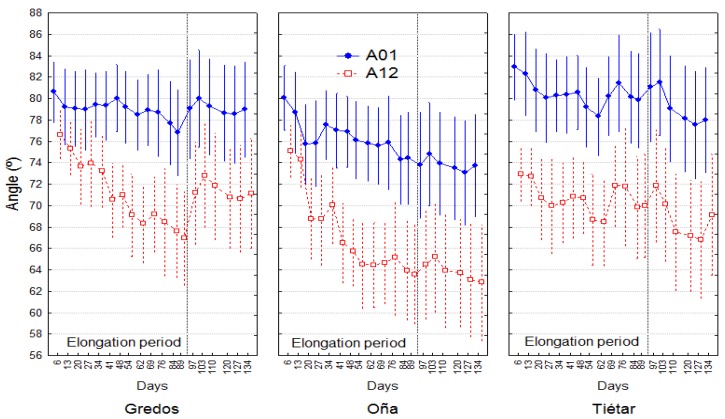
Changes over time in angles A01 and A12 in the three provenances under LLT: Dots represent least squares means, and bars represent 95% confidence intervals. Total number of observations n = 20.

In LLT, significant differences among provenances in angle A12 were observed (Figure 1 and Figure 6). A12 in Oña plants was 6.77° lower than in Gredos plants at the end of the experiment and 8.5° lower than in Tiétar plants. This total result was obtained from a combination of two processes: A) In the elongation period, Oña and Gredos plants reached a greater inclination (8.52° and 6.7° lower A12 than the Tiétar plants, respectively), suggesting a greater phototropic reaction in the plants from these two populations, with no significant differences between Gredos and Oña plants. B) In the second period (not elongation period), Gredos plants underwent a straightening process (increasing A12), while Oña plants continued to lean (Figure 6). The increase in A12 in Gredos plants in this period was significantly greater than that in both Oña (4.94° greater) and Tiétar (4.97° greater) plants. This enhanced straightening process in Gredos plants was also significant in A01, reaching 2.87° more than in Oña and 3.95° more than in Tiétar plants (Table 2).

### 2.3. Compression Wood

**Figure 7:**
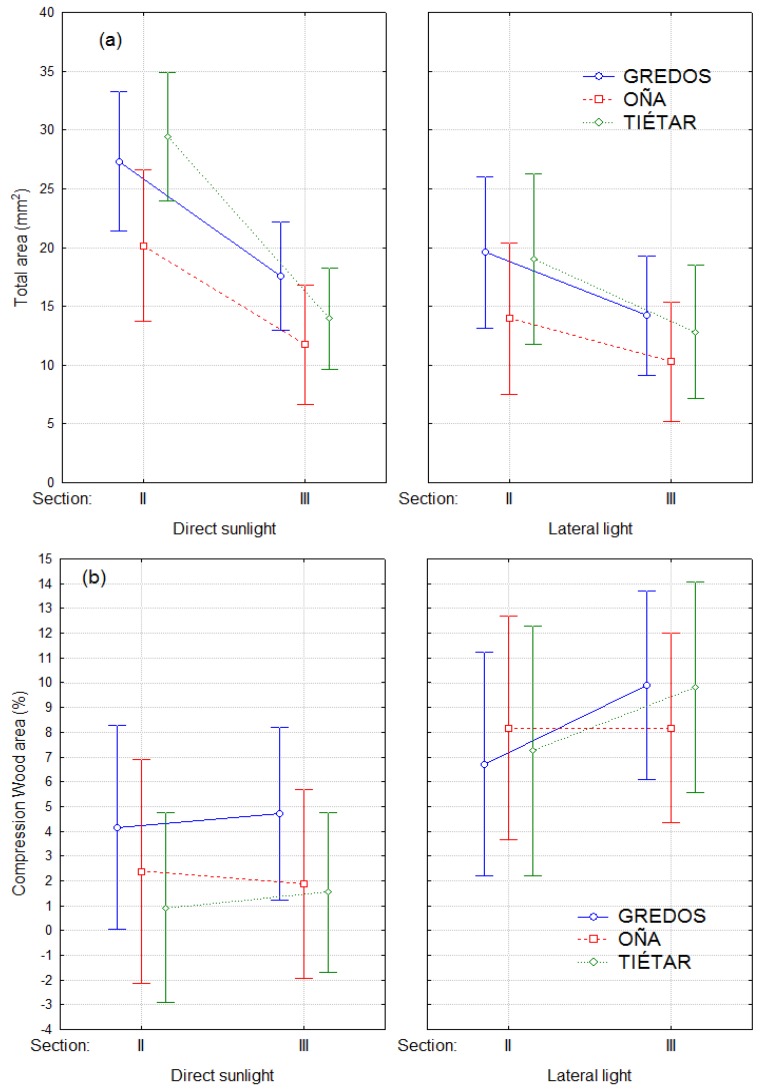
(**a**) Total area of cross-sections II and III by treatment and provenance. (**b**) Percentage of compression wood (CW) area with respect to the total area of cross-sections II and III per treatment (LLT and DLT) and provenance. Bars represent 95% confidence intervals. Total number of observations n = 36.

There were significant differences between treatments in the total area of the cross-section, CW area and percentage of CW area with regards to the total area, both in section II and section III, but there were no differences among provenances (Figure 7a).

Although CW was generally present in both treatments, it was greater in LLT than in DLT (Fisher’s exact test, *p* = 0.0426). In DLT plants, CW extended in 2.6% of the total cross-section area as a mean, whereas it occupied 8.3% of the cross-section in LLT (Figure 7b). In LLT, all the trees developed CW in some of the 4 studied cross-sections, while in DLT, 5 out of 18 trees did not show CW in any of them (3 plants from Tiétar, 1 from Oña and 1 from Gredos). In other terms, 87% of the sections studied showed CW in LLT versus 51% in direct sunlight.

In plants from LLT, CW was located mainly on the south side of the stem (71% of the cross-sections), that is, the lower side with respect to the leaning due to the phototropic reaction. In a few cases, the CW sector was located on the lateral sides of the stem (2% on the east and 5% on the west) but never on the north (upper side). The distribution of CW in the cross-sections of DLT plants was more balanced (17%, 4%, 14%, 16% on the south, north, east and west, respectively).

**Figure 8:**
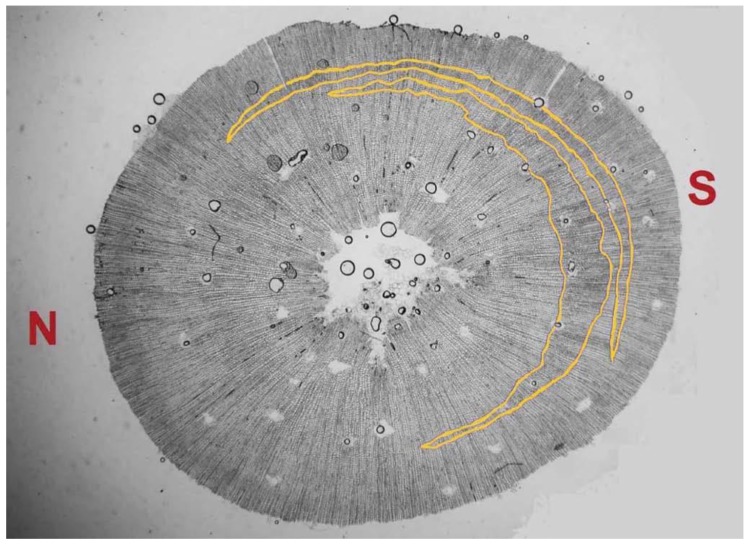
Concentric bands of CW in a cross-section of an LLT plant. N: north; S: south.

In all cases, the second ring of the cross-sections of LLT started developing normal wood (NW) before CW appeared. Frequently, CW in LLT appeared in two or more concentric bands alternating with NW (see Figure 8). These alternating bands were not observed in DLT plants.

## 3. Discussion

### 3.1. Photomorphogenetic Responses

Several factors were involved in the different environments experienced by the plants: The shade cloth caused changes in the quality, intensity and orientation of the light due to the shade cloth, which in turn reduced the temperature, evapotranspiration and mechanical wind effect. As a result, the elongation period was 20 days longer under LLT.

Different strategies to capture light and achieve mechanical stability have been reported at the species level [13,40] and have been confirmed to be related to individual plant fitness [40]. Different strategies may also exist at the population level, especially in species with high genetic variability and a wide distribution range, such as *P. pinaster*. As *P. pinaster* is a shade-intolerant species, the reduced intensity of the light can be assumed to be an important stressor to which different populations can react differently. In our experiment, LLT elicited differences in length growth among provenances that did not appear in DLT. In contrast, radial growth decreased under LLT conditions but without differences among populations. Plants from Tiétar provenance showed greater elongation than Gredos and Oña plants in the LLT as well as enhanced slenderness (both typical shade-avoidance responses), while there were no differences under direct sunlight, showing intraspecific variability at the population level in their responses to shade and, in particular, greater photomorphogenetic sensitivity in Tiétar plants.

### 3.2. Phototropic and Gravitropic Responses

The angle of inclination of the stem was significantly affected by LLT only in the growing part of the stem (A12) (see Figure 5), which was consistent with the observation that phototropic reactions appeared only in the elongating axes. An indirect effect on the leaning of the basal part (A01) can be expected due to the bending moment caused by the growth and the leaning of part 12, but this effect was not significant.

The small but significant asymmetry in radial growth between the north and south sides of the stem can be explained because all the plants were slightly inclined to the south at the beginning of the experiment. Additionally, the lack of effect of the lateral light treatment on A01 involves a lack of differences in asymmetry of the radial growth between treatments.

Although in this experiment it is not possible to disentangle the phototropic and gravitropic effects on leaning, the position of the stem described by angles A01 and A12 indicates the balance between them. In addition, we can assume that primary phototropic reactions occur only in the elongation period, while secondary gravitropic reactions likely occur throughout the experiment. Zhang et al. [41] observed in 6-year-old loblolly pines that CW was already formed in the lower part of the stems three days after tilting by 45°.

In LLT, Oña and Gredos plants reached a greater inclination (lower A12) than Tiétar plants in the elongation period, suggesting that Oña and Gredos plants are more sensitive to phototropic effects or that in the balance between photo- and gravitropic effects, the gravitropic effect is relatively weaker than the phototropic effect. When elongation ceased, the Gredos plants recovered verticality to some degree (increasing A12), while the Oña plants continued to lean. The recovery of verticality during the nonelongation period was significantly greater in Gredos plants than in the other two populations in both the basal and upper parts of the plants (A01 and A12). This result is consistent with a greater ability of Gredos plants to straighten after tilting, as shown by Sierra-de-Grado et al. [32]; these authors found higher maturation strains in tilted Gredos plants than in Tiétar and Oña plants, calculating the effect of CW on stem straightening with a biomechanical model proposed by Fournier et al. [34].

CW allows the tree to find a more favorable position and is usually associated with changes in stem orientation. In our experiment, most plants under LLT showed CW on the south side of the stem (lower side); the CW sector was located on the lateral sides of the stem in a few cases but never on the upper side. This finding means that the triggering of this CW is a gravitropic reaction to counter the leaning and not a way for the tree to move toward the light (phototropic). Both results, the lack of differences in asymmetry of the radial growth between treatments and the finding CW is not a mechanism for the movement of stems toward the light, indicate that these secondary reactions do not play any role in the phototropic responses.

The concentric bands of CW alternating with NW in LLT (Figure 8) could be related to the fluctuations observed in the leaning angles of each individual plant. These fluctuations may be the result of a balance between the phototropic and the gravitropic reactions (as well as to changes in the passive bending moment due to elongation growth) induced by the leaning of the growing stem toward the light. This phenomenon indicates that a gravitropic response would be triggered beyond a threshold of inclination, which may be variable in different individual plants or populations.

Herrera et al. [42] reported an alternative priority in the gravi- and phototropic reactions of *P. pinaster* seedlings under different levels of tilting and simultaneous lateral illumination: The plants tilted 30° or more had an immediate vertical primary reaction and then turned toward the light, while the plants tilted 0° and 15° directly turned to the light. In our experiment, a vertical reaction of the apex (primary gravitropic response) did not occur, but fluctuations of the leaning angles could respond to the alternative priorities involving both primary and secondary reactions.

The position of the CW bands within the ring after a zone of NW is consistent with this idea. During the elongation period, the stems started curving toward the light by primary reactions (differential elongation) while developing NW, and then when the leaning of the stem crossed a threshold, secondary reactions (with the development of CW) occurred until the excessive leaning had been counteracted and ceased when the photogravitropic equilibrium was restored. When the elongation period was finished, the leaning could again increase due to the increase in the bending moment caused by the growth of the needles, which could again trigger CW development. This explanation involves only primary photo(gravi)tropic and secondary gravitropic responses. We have not found evidence of secondary phototropic reactions, allowing us to reject the involvement of CW as a motor of secondary phototropic responses in this experiment.

### 3.3. Are the Differences Related to Stem Straightness?

Our hypothesis was that differences in stem straightness among populations can be related to different sensitivities to external stimuli that induce curvature, in this case, light conditions. The straighter population, Gredos, exhibited a response that was very similar to that of the twisted Oña in terms of low photomorphogenetic and high phototropic responses during the elongation period. Hence, we can reject the hypothesis that a lower sensitivity to lateral light plus shade is related to improved straightness at the population level in our experimental conditions. However, new experiments with a wider range of light quality and intensity could add valuable information about intraspecific variation, such as the ability to detect changes in light intensities and spectral variations that allow plants to detect neighbors and lead to SAS responses.

In contrast, the straighter population (Gredos) showed a greater ability to straighten than either of the twisted populations (Oña and Tiétar). There were no significant differences among provenances in the CW area or in the percentage of CW area with regard to the total area, suggesting that the greater ability of Gredos plants to straighten may be related to more efficient rather than to more extensive CW development; in other words, with the same amount of CW, a greater correction of the curvature is achieved. This finding is also consistent with the results of previous tilting experiments with plants from the same populations [32].

The differences among provenances in leaning angles and growth traits were detected only under LLT conditions, that is, under a particular stimulus causing curvatures in the stem. Sierra-de-Grado et al. [12,43] reported a dramatic increase in the heritability of apical and global leaning of the stem in plants under increasingly stressful environments due to changing light conditions with respect to the induction of curvature in the stem. These findings reinforce the idea that selection processes for straightness in breeding programs would be more efficient under conditions of inductive “mechanical stress” (conditions for curving the stems). In particular, the efficiency of the gravitropic correction seems to be the main driver of stem straightness in adult stages and can be revealed only through specific inductive experiments [14,32].

## 4. Materials and Methods

### 4.1. Plant Material

Twenty regions of provenance in Spain have been defined based on geographical distribution and isolation, ecological conditions and administrative limits. Three regions (Gredos, Tiétar y Oña) were chosen due to their differences in stem straightness and growth, as described in the introduction.

The pine woodlands of Sierra de Gredos provenance cover 39,200 ha, at altitudes between 500 and 1500 m a.s.l. The soils are developed on granite and are quite homogeneous in composition, mostly sandy and moderately acidic. The annual rainfall ranges from 648 mm in the Northeast to 1640 mm in the Southwest. Trees from this provenance show excellent straightness and growth, and those characteristics are maintained in provenance trials outside their place of origin [6,7]. Bajo Tiétar provenance is composed of several small populations close to the river Tiétar. Its altitude is lower than 400 m a.s.l. Its soils are moderately acidic, developed on quartz sands. The annual rainfall is 1060 mm. The trees show good growth, but the stems typically have a corkscrew form [5]. The Sierra de Oña population occupies 15,000 ha in north central Spain, in an altitude range between 600 and 1200 m. This region is heterogeneous in terms of soil, with calcareous and siliceous areas. The annual rainfall is 528 mm. This population shows slow growth, high production of cones and great bark thickness. The quality of the logs is very low due to the frequent and pronounced curvature [5,7].

Seeds from trees of the three populations were sown in square-based pots. The seedlings were grown for one year in a greenhouse covered by a shade cloth. In the second spring, the pots were placed outdoors on iron structures specially designed for the experiment to keep the pots in a fixed position. Due to growth in the greenhouse for the first year, all the stems had a slight inclination before starting the experiment. They were placed with the inclination toward the south in two parallel complete blocks in the east-west direction. Two treatments were established: Direct sunlight (DLT) and lateral light plus shade (LLT).

### 4.2. Measurements and Statistical Analysis

Throughout the experiment (from the beginning of May to mid-September), each plant was photographed weekly, with the camera in the same place every week and each photograph taken of the north-south plane. Photographs of the north-south plane recorded the main changes in the plants in response to lateral light coming from the south.

To analyze the changes in the leaning of the stems, the angles of segments 01 and 12 of the stems (Figure 1) with respect to the horizontal direction were measured in the photos (A01 and A12, respectively). The stem length was also measured based on the pictures, which included a scale. Length growth was computed as L_i_−L_0_, with L_i_ being the length i days after the beginning of the experiment and L_0_ the initial length. At the end of the experiment, the difference between the mean length growth under LLT and the mean length growth in DLT of the same provenance provided an estimation of the mean photomorphogenetic effect at the population level. All measurements were performed using Photoshop 5.5 and 6.0 (Adobe Systems Incorporated, 2000).

Statistical analysis of the length growth and angles was performed using repeated-measures ANOVA with two between-subjects factors (treatment and provenance) in a 2 × 3-factorial design with two blocks with covariables L0 (initial length) for length growth and A0 (initial angle of segment 01) and L0 for angles. We used a linear mixed model with one within-subject factor (days) for length growth and a general linear model with two within-subject factors (segment and days) for angles. Individual contrasts were performed for all the comparisons. The analyses were performed with Statistica ’99 Edition and SAS 9.4. The initial number of plants per provenance and treatment was 10, but due to the death of some plants during the experiment, the number of plants used for the analysis was 28 under DLT (9 from Gredos, 9 from Oña and 10 from Tiétar) and 20 under LLT (7 from Gredos, 7 from Oña and 6 from Tiétar).

At the end of the experiment, 18 plants from each treatment were defoliated, marked on the south and west sides, and then harvested. The numbers of plants by provenance were 6 from Gredos, 5 from Oña and 7 from Tiétar in DLT and 7 from Gredos, 5 from Oña and 6 from Tiétar in LLT. Cross-sections were obtained at the base of the stem (section I), immediately above the cotyledons (section II), at the middle (section III) and at the end (section IV) of segment 01 of the stem (Figure 1).

In section II, the radial growth of the second ring to the north and to the south and the total diameters in the north-south and east-west directions were measured. The slenderness was computed as the final length of the plant (cm) divided by the mean total diameter in section II (mm).

The cross-sections were analyzed microscopically to determine the presence of CW based on the anatomic features of the tracheids: A more rounded cross-sectional profile than that of NW tracheids and a thicker cell wall [3,4,44,45]. The thickness of the cross-sections was between 12 and 16 μ, obtained using a microtome, with an angle of incidence of the blade of 9°. The sections were placed in slides with glycerin. The total area of the sections and CW area were measured with a planimeter on printed calibrated images of the sections and analyzed by repeated-measures ANOVA, with treatments and provenances as between-subjects factors and section as the within-subjects factor. Individual contrasts were performed for all comparisons.

## Figures and Tables

**Table 1:** Comparison of length growth under DLT and LLT by provenance. L0: Mean initial length (cm) of the plants; Gd and Gl: Final length growth (cm) in DLT and LLT, respectively. Means without any common letter are different at the 5% significance level.

Provenance	DLT			LLT			Gl − Gd	Gl/Gd %
	L0	Gd = L134 − L0		L0	Gl = L134 − L0			
GREDOS	21.97 a	6.51 a		21.32 a	20.51 ab		14.00 b	315%
OÑA	16.54 b	5.38 a		17.95 b	18.78 b		13.40 b	349%
TIÉTAR	24.67 a	4.80 a		23.04 a	24.78 a		19.98 a	516%

**Table 2:** Individual contrasts between provenances for the variation of angles A01 and A12 in both treatments. The contrasts were conducted in three periods: (a) from the beginning to the end of the experiment (*Total*), (b) *Elongation period* (from the beginning of the experiment to 69 days in DLT and to 89 days in LLT), and (c) *Not elongation period* (from the end of the elongation period to the end of the experiment). Ls-means (°) and (*p*-value) are shown. Bold, significant differences.

*Treatment*	Contrast	*Total*			*Elongation Period*			*Non-Elongation Period*	
		∆ A01	∆ A12		∆ A01	∆ A12		∆ A01	∆ A12
DLL	Gredos−Oña	2.47	3.96		1.04	2.96		1.43	1.00
		(0.3923)	(0.2112)		(0.6219)	(0.2361)		(0.3745)	(0.6469)
	Gredos−Tiétar	0.54	1.71		−1.48	−0.1281		2.02	1.84
		(0.8383)	(0.5572)		(0.4514)	(0.9556)		(0.1815)	(0.3652)
	Oña−Tiétar	−1.92	−2.25		−2.51	−3.09		0.59	0.84
		(0.5615)	(0.5344)		(0.3023)	(0.2823)		(0.7507)	(0.7360)
LLT	Gredos−Oña	4.69	**6.77**		1.83	1.83		**2.87**	**4.94**
		(0.1235)	**(0.0441)**		(0.5035)	(0.5136)		**(0.0202)**	**(0.0173)**
	Gredos−Tiétar	3.32	−1.73		−0.63	**−6.70**		**3.95**	**4.97**
		(0.2692)	(0.5950)		(0.8172)	**(0.0205)**		**(0.0019)**	**(0.0162)**
	Oña−Tiétar	−1.37	**−8.50**		−2.45	**−8.52**		1.08	0.03
		(0.6739)	**(0.0214)**		(0.4099)	**(0.0078)**		(0.4036)	(0.9900)
